# Hydrogen-bonded multi-mode liquid crystal elastomer actuators†

**DOI:** 10.1039/d4tb02228a

**Published:** 2024-12-18

**Authors:** Roshan Nasare, Hongshuang Guo, Arri Priimagi

**Affiliations:** a Smart Photonic Materials, Faculty of Engineering and Natural Sciences, Tampere University Tampere P.O. Box 541 FI-33101 Finland hongshuang.guo@tuni.fi arri.priimagi@tuni.fi +358 0468415232 +358 0445150300

## Abstract

As biomimicry advances, liquid crystal elastomers (LCEs) are gaining attention for their (multi-)stimuli-responsiveness and reversible shape morphing. Introduction of dynamic bonds into the LCEs provides versatile means towards programmable shape morphing and adaptation to environmental cues, and new designs for dynamic LCEs are actively sought for. Here, we present a supramolecular LCE that integrates shape memory programming, humidity sensitivity, and photochemical actuation. By utilizing hydrogen bonding crosslinks, the LCE gains shape memory functionality, enabling arbitrary shape programming and photochemical actuation. By breaking the supramolecular crosslinks *via* base treatment, the LCE becomes hygroscopic and humidity sensitive, yet maintains photochemical deformability. These two states enable different types of soft actuator demonstrations both in air and under water, adding to the versatility and programmability of light-driven shape-changing LCEs.

## Abstract 2

We present multi-mode, supramolecular liquid crystal elastomer actuator that integrates shape memory programming, humidity sensitivity, and photochemical actuation, depending on the pre-activation conditions.

## Graphical abstract

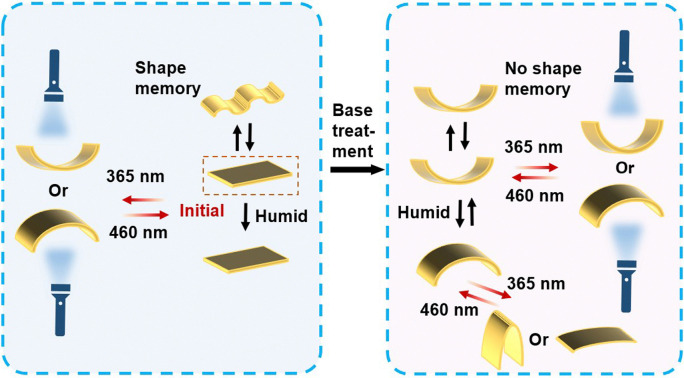

## 1. Introduction

Liquid crystal elastomers (LCEs) are a unique class of stimuli-responsive soft materials that combine anisotropic molecular order and elasticity.^1,2^ This combination may lead not only to tunable optical properties,^3,4^ but also to large, reversible shape changes in response to various stimuli such as temperature,^5–7^ electric^8^ and magnetic fields,^9^ and light.^10^ The shape changes are rooted in external control over the molecular alignment that can be pre-programmed during the sample fabrication *via*, *e.g.* surface-enforced alignment or shear forces. Due to their unique properties, LCEs bear significant potential in soft robotics,^10,11^ photonics,^12^ and biomedical technologies.^13,14^ In many of these applications, light is a desirable stimulus for triggering the LCE shape changes, enabling remote control with high spatiotemporal precision. The light-driven shape changes can be obtained either photothermally by adding light-absorbing dyes or nanoparticles into the LCE,^15–17^ or photochemically *via* incorporation of molecular switches such as azobenzenes,^18^ hydrazones,^19^ or donor–acceptor Stenhouse adducts.^20^ The photochemical actuation mechanism is particularly attractive when stable shape changes or underwater functionalities are desired.

In addition to their inherent temperature-responsiveness, most LCEs respond to a single stimulus in a manner dictated by their initial molecular alignment. While examples of reconfigurable LCEs exist,^21–23^ multifunctional LCEs with programmable shape morphing ability or adaptation to environmental cues are actively sought for. Such systems open diverse opportunities in bioinspired soft robotics^10,24^ and in emulating complex biological processes such as dynamic self-assembly,^25,26^ self-healing^27^ or learning.^28^ From a more technological perspective, multifunctional LCEs or LCE assemblies may find use in, *e.g.*, smart textiles, soft electronics, or as adaptive soft actuators and sensors.

One prevalent strategy in creating multifunctional LCEs involves incorporating dynamic bonds into the LCEs. Dynamic covalent bonds enable properties like reprogramming, reprocessability, and recyclability.^29,30^ Supramolecular interactions such as hydrogen bonding in turn, can be used as chemically responsive (*i.e.*, pH-responsive) or as dynamic structural (*i.e.*, temperature-responsive) moieties, yielding stimuli-induced changes in shape, colour, porosity or selective binding.^31–34^ Among the different hydrogen-bonding motifs, dimerization of carboxylic acids has been particularly widely explored.^35,36^ Dimers of benzoic acid are mesogenic and easy to incorporate into the LCEs. The dimers can be broken through alkaline base treatment, to yield a hygroscopic carboxylate salt that renders the LCE humidity responsive and enables reversible actuation driven by anisotropic swelling/deswelling.^37^ Carboxylic dimers may also be utilized as supramolecular crosslinks, establishing connections between polymer chains that break at high temperatures and reform upon cooling. Such dynamic crosslinks give rise to a shape memory effect, enabling LCEs that combine shape memory programming and reversible actuation.^38^

In this work, we aim at combining the aforementioned feats of carboxylic-acid-containing LCEs to devise a photochemical actuator system that can undergo a shape memory effect or that can be sensitized to humidity – depending on the pre-activation conditions. The former is enabled by utilizing the carboxylic acids as supramolecular crosslinks, and the latter by converting them into carboxylic salts. As we will show, the shape programming and shape morphing properties of the resultant LCEs are very distinct in these two states, enabling different types of soft actuator demonstrations and adding to the versatility and programmability of light-driven shape-changing LCEs.

## 2. Results and discussion

**Fig. 1:**
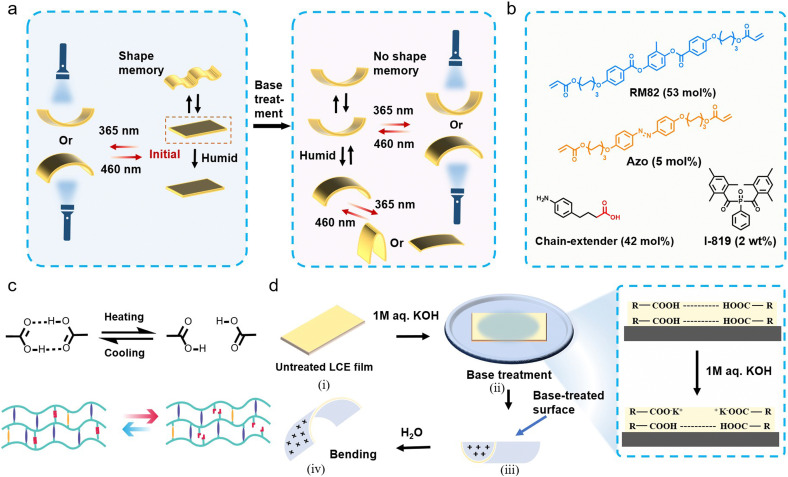
Material design concept. (a) Schematic illustration depicting a photochemically driven, humidity-sensitive LCE actuator with shape-programming and shape-morphing properties. (b) The chemical composition of the LCE. (c) The shape-memory programming relies on the breaking and reforming of hydrogen bonds upon heating and cooling, respectively. (d) The humidity sensitization process: (i) a planar-aligned LCE film attached to a glass substrate is (ii) immersed in 1 M KOH solution to asymmetrically break the hydrogen bonds and yield a sample that is saltified from one side of the film. (iii) Base treatment causes shrinkage of the film, resulting in an increase in curvature. (iv) Bending of the activated film in a humid environment.

Our materials design concept, depicted in Fig. 1a, targets a multifunctional LCE with a distinct shape-morphing ability depending on pre-activation conditions. The compounds used, and their stoichiometry, is shown in Fig. 1b. More specifically, we aim at devising a photochemically actuable LCE crosslinked *via* dynamic supramolecular bonds that break upon heating and re-form upon cooling (Fig. 1c) and enable shape programming *via* shape memory effect.^38^ On the other hand, the carboxylic acid groups also act as handles to sensitize the LCE to humidity upon base treatment,^39–41^ to yield a photochemically driven system that can orthogonally respond to light and environmental humidity (Fig. 1d). Ideally, the LCE could be switched back and forth between the two states, depending on the desired use case.

To obtain this, we prepared chain-extended LCEs *via* Aza–Michael addition reaction,^42–44^ using carboxylic-acid-functionalized, amine-based chain extenders that can form hydrogen-bonded dimers and allow – when desired – subsequent activation *via* base treatment. The chain extender was chosen (i) to be non-mesogenic so that breaking the hydrogen bonds would not disrupt the molecular alignment, and (ii) to have a lower melting point than the carboxylic-acid-functionalized alkylamine we used in our earlier studies (*ca.* 120 *vs. ca.* 170 °C),^38^ to enable easier processing. The reaction mixture comprises the widely used diacrylate mesogen RM82 in combination with 4,4′-bis[6-(acryloyloxy)hexyloxy]azobenzene (Azo) to render the LCE photochemically active in response to UV/visible light. The experiments have been carried out with planar-aligned LCEs with a thickness of 100 μm, except for some UV-vis characterizations conducted with 20 μm samples (see Materials and methods for further fabrication details).

**Fig. 2:**
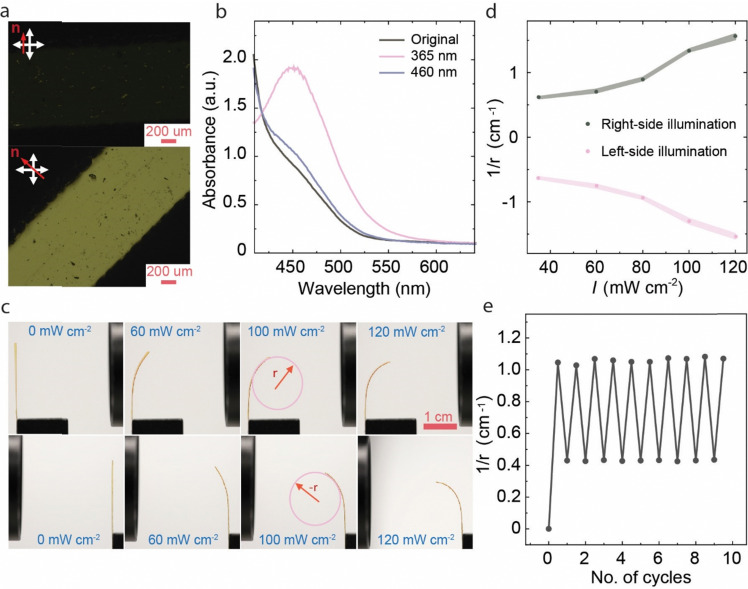
Photochemical actuation before base treatment. (a) Polarized optical micrographs of the LCE at 0° and 45° between the molecular director and the polarizer/analyzer, demonstrating uniaxial molecular alignment. (b) Unpolarized UV-vis spectra (at the n–π* absorption band) of the planar-aligned LCE (thickness 100 μm) in the dark, after irradiation with 365 nm (15 mW cm^−2^, 30 s), and after subsequent irradiation with 460 nm (25 mW cm^−2^, 30 s). (c) Photographs taken after UV irradiation at different light intensities from both sides of the sample, depicting symmetric photochemical deformation of the LCE. *r* and −*r* correspond to curvatures during bending to the right and left side, respectively. (d) Corresponding curvature measurements show symmetric bending of the LCE film in the direction of the UV-light source, no matter from which direction it is illuminated. (e) Bending and unbending under 365 nm (90 mW cm^−2^, 10 s) and subsequent 460 nm (200 mW cm^−2^, 10 s) irradiation over 10 cycles.

Fig. 2 illustrates the characteristics of the pristine LCE before base treatment. The planar alignment of the film was confirmed *via* polarized optical microscopy, as depicted in Fig. 2a, where distinct light and dark variations were observed upon rotating the sample under the polarizer. The order parameter of the films was in the range of 0.45, as estimated for 20 μm films polymerized in an identical manner as the 100 μm films (Fig. S1a, ESI†). The planar-oriented LCE exhibited reversible deformation in response to temperature changes (Fig. S2, ESI†) and underwent 12% contraction along and 14% expansion perpendicular to the director upon heating to 200 °C. Reversible isomerization of the azobenzene molecules was ensured by monitoring the n–π* absorption band at 450 nm (the higher-energy π–π* band was too intense to be monitored in the 100 μm samples), which intensified upon 365 nm illumination due to *trans*–*cis* isomerization and restored the initial absorbance upon subsequent 460 nm irradiation due to *cis*–*trans* isomerization (Fig. 2b).

Under 365 nm illumination, the planar-aligned free-standing LCE strip bent towards the light source (Fig. 2c) due to the *cis*-isomer gradient formed through the sample thickness.^45,46^ The sample retained the bent state after ceasing the illumination and reverted to the initial state under 460 nm irradiation, as characteristic of photochemically induced actuation. The bending is symmetric, *i.e.*, the curvature obtained is the same no matter from which side the strip is illuminated, as expected for planar-aligned LCEs. The deformation was intensity-dependent, with higher intensity leading to higher bending curvature (Fig. 2d and Fig. S3a and b, ESI†). At high irradiation intensities, the sample also experienced photothermal heating (Fig. S4, ESI†), causing partial relaxation of the deformed state after ceasing the UV irradiation. As shown in Fig. S4d (ESI†) the photothermal effect was significantly boosted by using both UV and visible illumination simultaneously, *i.e.*, when driving the azobenzene molecules to cyclic motions between the *trans* and *cis* isomers. The photochemical bending/unbending cycles were reversible over at least 10 irradiation cycles, as shown in Fig. 2e.

**Fig. 3:**
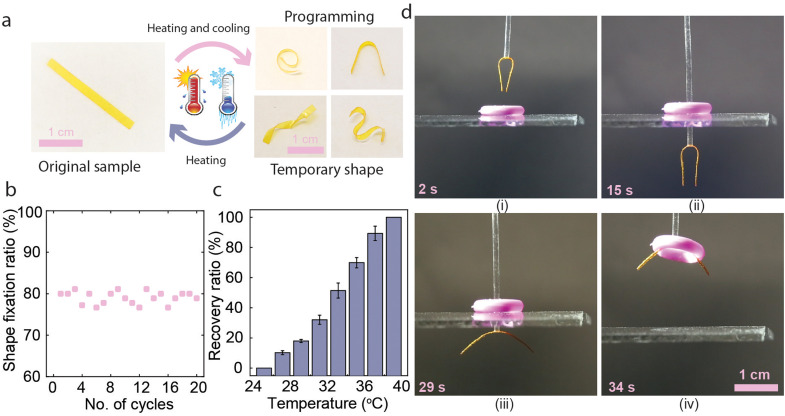
Shape memory programming of untreated LCE. (a) The process involves mechanical deformation at 70 °C followed by cooling to room temperature under mechanical load. The original flat shape could be retained upon re-heating. (b) Shape fixation ratio after multiple heating and cooling cycles. (c) Shape recovery ratio of the LCE at different temperatures shows that the original shape is fully restored at around 40 °C. (d) Light-controllable “gripper” utilizing the shape memory effect: (i) LCE is shape-programmed to a bent shape enabling it to pass through an object with a circular hole. (ii) Irradiation with 385 + 460 nm (150 + 100 mW cm^−2^, 20 s) and resultant temperature increase (iii) cause the LCE to return to its original shape, after which (iv) the object can be lifted.

In addition to photochemical actuation ability, the pristine LCE exhibited shape memory effect and could be programmed to various temporary shapes (Fig. 3a). This can be explicated by the presence of the dynamic carboxylic acid dimers that are broken at elevated temperatures and reform upon cooling^47–50^ (Fig. 1c). We confirmed this by FTIR spectroscopy (Fig. S5, ESI†), showing that the *ν*(C=O) peak appearing at 1724 cm^−1^ shifts to 1726 cm^−1^ as the temperature increases from 30 to 90 °C, indicating the breaking of hydrogen bonds.^51^ The FTIR data indicates that the hydrogen bonds begin to dissociate at around 60 °C. Hence, we selected 70 °C as the shape programming temperature. We then measured the shape fixation ratio after multiple heating and cooling cycles for different shapes, and it remained around 80% over at least 20 cycles, as shown in Fig. 3b. The shape recovery ratio at different temperatures is shown in Fig. 3c. The temporary shape is stable at 25 °C, but the film gradually recovered at higher temperatures and reached the original shape at around 40 °C (Fig. S6, ESI†). The pristine LCE can, *e.g.*, be used to move circular objects in a manner depicted in Fig. 3d (see also Movie S1, ESI†). The process starts by gluing an LCE strip with an initially flat shape to a vertical tube and shape-programming it into a bent state (i) to fit through the central hole of an object being lifted (ii). Next, the initial flat shape is restored *via* photothermal heating (iii) to lift and move the object (iv). Finally, the object can be released at a desired location, either mechanically or *via* photochemical actuation. The gripper also functions in an underwater environment, as demonstrated in Fig. S7 and Movie S2 (ESI†).

**Fig. 4:**
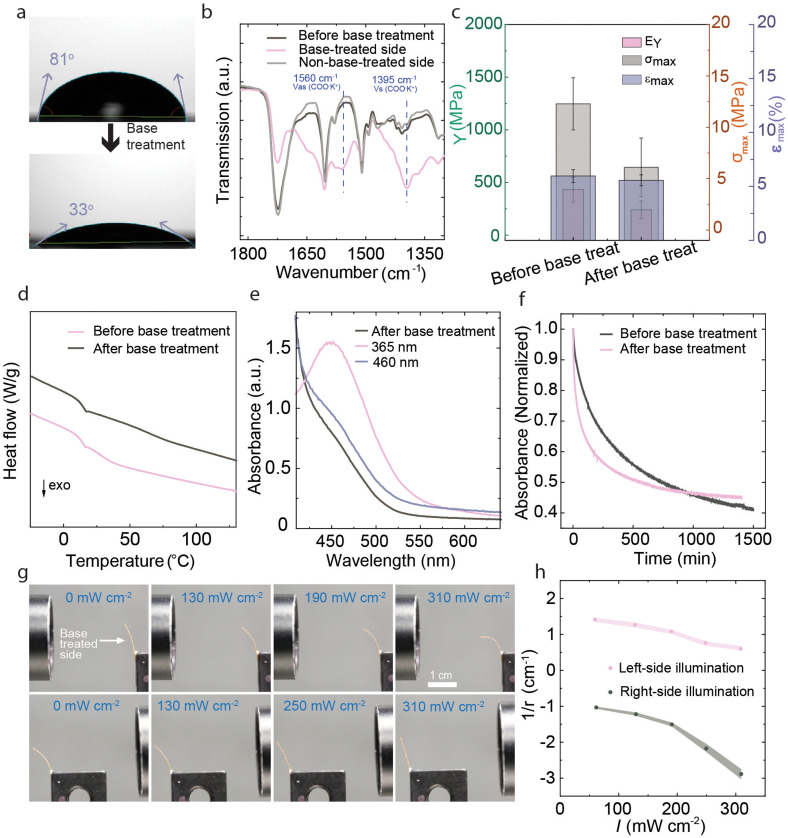
After base treatment. (a) Contact angles, (b) FTIR spectra, and (c) Young's modulus (*E*_Y_), fracture strain (*ε*_max_), and tensile strength (*σ*_max_) of the LCE before and after base treatment. The error bars represent the standard deviation obtained from *n* = 3 experiments. (d) DSC curves before and after base treatment, measured during the second cooling cycle with a rate of 10 °C min^−1^. (e) The n–π* absorption band of the base-treated LCE (thickness 100 μm) in the dark, after irradiation with 365 nm (15 mW cm^−2^, 30 s), and after subsequent irradiation with 460 nm (25 mW cm^−2^, 30 s). (f) Thermal *cis–trans* relaxation curves (monitored at 480 nm) measured from a 20 μm thick LCE before and after base treatment. (g) Photographs of photochemical deformation of the base-treated LCE after UV illumination (different intensities) from either side, under 35% relative humidity, and (h) corresponding bending curvatures.

The pristine LCE could be photochemically actuated and shape-programmed into desired shapes *via* the shape memory effect. However, it did not respond to changes in relative humidity. For humidity sensitization, we followed the methodology developed by Schenning and coworkers^39,40^ and treated one side of the 100 μm thick LCE with 1 M KOH solution (Fig. 1d) for 10 min to break the hydrogen-bonded dimers to yield a carboxylate salt. This led to significant changes in the hydrophilicity of the LCE, the water contact angles being 81° and 33° before and after the base treatment, respectively (Fig. 4a). ATR-FTIR spectra (Fig. 4b, see Fig. S8 for the full FTIR spectra, ESI†) showed the lowering of the hydrogen bonding peak at 1724 cm^−1^ and the appearance of two new peaks at 1560 cm^−1^ and 1395 cm^−1^, corresponding to COO^−^ stretching on the base-treated side of the film.^39^ No spectral changes were observed on the opposite side of the film, confirming asymmetric base treatment and the formation of salt gradient through the thickness of the LCE. Polarized optical microscopy (Fig. S9, ESI†) indicated that the LCE retained the planar molecular alignment also after base treatment. To quantify the molecular alignment further, we measured the order parameter of 20 μm samples, which we assume, under a similar base treatment procedure, to be saltified throughout the sample thickness. As shown in Fig. S1b (ESI†), practically no changes were detected in the order parameter before and after base treatment, which we attribute to the use of non-mesogenic carboxylic acid dimers as opposed to the benzoic-acid-based supramolecular mesogens.^36,37^ As shown in Fig. 4c and Fig. S10 (ESI†), Young's modulus and tensile strength before base treatment (432 MPa/12.1 MPa) were higher than after the base treatment (240 MPa/6 MPa), and the corresponding fracture strain increased from *ca.* 4.1% to 5.2%. On the other hand, no significant change was observed in the glass transition temperature (*T*_g_, Fig. 4d), which is *ca.* 12 °C before and after base treatment. We note, however, that the thermomechanical analysis is done for samples that are only partially saltified, *i.e.*, the comparison is not to be taken between fully crosslinked and fully saltified cases.

The base treatment did not significantly affect the UV-induced *trans–cis* isomerization or the reverse isomerization in response to blue light (*cf.*Fig. 2b and 4e). However, the thermal *cis*–*trans* isomerization kinetics, measured from pristine and base-treated LCEs with 20 μm thickness, are drastically different. As shown in Fig. 4f (see also Fig. S11, ESI†), the thermal half-lives are *ca.* 232 min and 45 min before and after base treatment, respectively. The shorter lifetime can be caused by increased free volume due to breaking the hydrogen-bonded network, and potentially by polarity changes. Despite the shorter *cis*-lifetime, the base-treated sample retained its photochemical activity, bending towards the light source under UV illumination (Fig. 4g) and reverting to the original shape upon subsequent blue-light illumination. Unlike in pristine LCEs (Fig. 2d), the asymmetrically base-treated samples displayed an initial curvature due to changes in hygroscopicity and mechanical properties at the base-treated side. Due to the same reasons, the photochemical deformation became asymmetric. As shown in Fig. 4g and h (see also Fig. S12, ESI†), the base-treated LCE-strips still bent towards the light source, but the curvature changes were significantly more pronounced when the sample was irradiated from the base-treated side, as could be expected due to its mechanical softening compared to the non-treated side.

**Fig. 5:**
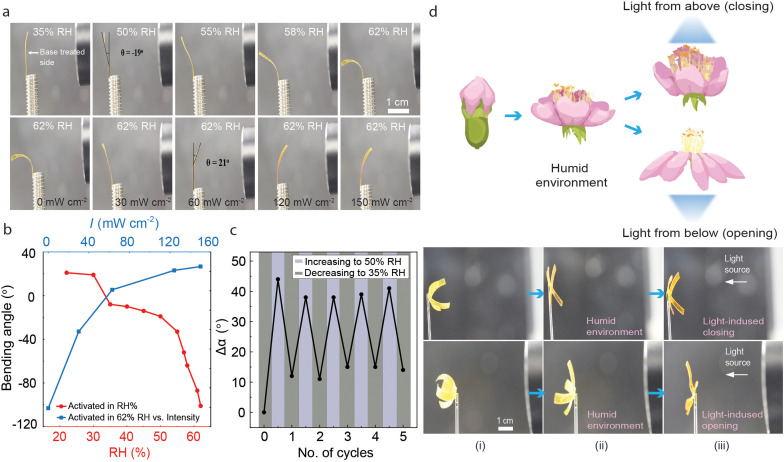
Light- and humidity-sensitized actuation after base treatment. (a) Deformation of the base-treated LCE in response to humidity (top) and UV irradiation under constant (62%) relative humidity (bottom). (b) Change in bending angle of base-treated LCE strips in response to increasing RH in the dark (blue line), and the photoinduced bending at 62% RH in response to increasing light intensity (red line). (c) Bending angle of the activated film upon exposure to 35% and 50% humidity for 5 cycles. (d) Top: Schematic illustration of a flower opening its petals in a humid environment and controlling its opening or closing with light. Bottom: Light- and humidity-responsive LCE structure made from base-treated LCE in a humidity-controlled chamber. (i) The structure, with base-treated sides facing inside, in RH 35% at room temperature. (ii) The petals open when the RH is increased to 62%. (iii) Upon irradiation with 365 nm, the actuation depends on irradiation direction and can lead either to light-induced opening or closing; light shines on activated and non-activated surfaces with different light intensities, allowing the flower petals to open and close further.

The base-treated LCE retained its photochemical activity, however, the shape memory properties were lost. Hence, a different set of soft actuation demonstrations can be accessed before and after the activation process. Relative humidity plays a significant role in the bending of base-treated samples.^37,39^ As shown in Fig. 5a (top), the LCE deforms systematically with increasing RH%, bending away from the base-treated side due to its increased hygroscopicity and resultant swelling. The humidity-induced bending is retained at least over a period of four months, as shown in Fig. S13 (ESI†). FTIR spectra of the aged, base-treated LCE still shows the lowering of the hydrogen bonding peak at 1724 cm^−1^ and the presence of carboxylate functional peaks at 1560 cm^−1^ and 1395 cm^−1^ at the base-treated side (Fig. S14, ESI†). Upon UV illumination at constant humidity, the strip still bent towards the light source irrespective of the illumination direction (Fig. 5a bottom). Bending and unbending of the activated film were examined through multiple cycles with changing RH, remaining relatively stable over at least 5 cycles (Fig. 5c). The interplay between humidity-driven and light-driven actuation of LCEs is multitude and depends on whether the photocontrol is photothermal or photochemical in nature. In the latter case, the humidity and light stimuli are coupled, and the photothermal heating causes the release of absorbed water and resultant deswelling of the base-treated side, enabling photoactuation that is gated by environmental humidity.^37^ In the case of photochemical actuation, the two control signals are orthogonal and the light response is triggered by isomerization-induced changes in LCE molecular alignment rather than swelling/deswelling. This can be utilized, *e.g.*, in devising a flower-like planar-aligned LCE structure (base-treated side facing “inside”) where the petals bend towards the same central point. Upon increasing humidity, the petals open due to water absorption (Fig. 5d). The orthogonal photocontrol is demonstrated by the fact that depending on irradiation direction, UV illumination causes the structure to either open or close, while blue-light illumination causes motion to the opposite direction. At the same time, and depending on the irradiation conditions, the sample can also be photothermally activated (Fig S4, ESI†), hence enabling switching between the orthogonal *versus* gated stimuli-responsiveness if desired.

## 3. Conclusion

We present a multi-mode liquid crystal elastomer (LCE) with distinct shape morphing ability, depending on pre-activation conditions. Switching between different states is enabled using carboxylic-acid-containing chain extenders, which can form dynamic supramolecular crosslinks or alternatively act as handles for saltification *via* base treatment. In the former state, the LCE is humidity-insensitive, yet exhibits shape memory properties. In the latter state, the shape memory properties are lost, but the LCE actuation can be driven by humidity changes due to anisotropic swelling. Due to the presence of azobenzene, both states are photochemically active, and they enable distinct soft actuator demonstrations both in the air and underwater. Further work is needed for reversible switching between the two states, but we believe our work lays the groundwork for multimodal shape-changing materials where dynamism is introduced by making and breaking supramolecular bonds.

## 4. Experimental section and methods

### 4.1. Materials

1,4-Bis-[4-(6-acryloyloxyhexyloxy)benzoyloxy]-2-methylbenzene (RM82, 99%), and 4,4′-bis(6-acryloyloxyhexyloxy)azobenzene (Azo) were purchased from SYNTHON Chemicals GmbH & Co. 4-(4-Aminophenyl)butyric acid, used as chain extender, was purchased from Merck. Phenylbis(2,4,6 trimethylbenzoyl)phosphine oxide (I-819) was purchased from TCI. All reagents and chemicals were used as received without further purification.

### 4.2. LCE film preparation

LC cells were formed from two glass substrates that were spin-coated (JSR OPTMER) with polyvinyl alcohol (PVA, 5% water solution, 4000 rpm for 1 min, baked at 100 °C for 5 min), rubbed uniaxially with a satin cloth to ensure planar LC alignment, and subsequently blown with high-pressure air to remove dust particles from the surface. The glass slides were glued together using UV-curable glue (UVS 91; Norland Products, Cranbury, NJ) to form the cell. While glueing, 20 μm or 100 μm microspheres (Thermo Scientific) were used as spacers to determine the sample thickness.

The LCE mixture was prepared by mixing 53 mol% RM82, 5 mol% Azo, 42 mol% chain extender, and 2 wt% I-819. The mixture was heated to 130 °C and mixed thoroughly by magnetically stirring (200 rpm) for 15 min. The stirred mixture was filled into the LC cells *via* capillary effect at 130 °C and cooled down to 63 °C with a rate of 3 °C min^−1^. The cells were kept at this temperature for 20 h to allow the Aza–Michael addition reaction to occur, followed by photopolymerization under UV + visible light (385 nm + 460 nm; 60 mW cm^−2^ and 25 mW cm^−2^, respectively) at 63 °C. The polymerization was conducted for 20 min from both sides of the LC cell. Finally, the cells were opened with a blade and strips with the desired dimensions were cut from the film for further characterization and experiments.

### 4.3. Base treatment of the film

The base treatment was conducted after photopolymerization by opening the LC cell while keeping the film on the glass substrate to ensure carboxylate salt formation from only one side of the film. The sample, having one side exposed to air and the other firmly attached to the substrate, was immersed in 1 M KOH solution for 10 min and dried with high-pressure air. Finally, the base-treated film was removed from the glass substrate for further use.

### 4.4. Instrumentation and material characterization

Fourier transform infrared (FTIR) spectra were recorded on a PerkinElmer spectrum two spectrometer between 4000–650 cm^−1^, resolution 2 cm^−1^ with a clean ATR crystal used as reference. Differential scanning calorimetry (DSC) measurements were performed with Netzsch DSC 214 Polyma at a heating/cooling rate of 10 °C min^−1^. The measurements were performed with 5–10 mg of sample at 1 bar under nitrogen atmosphere (flow rate of 20 mL min^−1^) in the temperature range between −50 and 150 °C. The stress–strain curves were determined with a homemade tensile tester from 100 μm thick films with a stretching speed of 0.05 mm s^−1^. The LED source CoolLED pE-4000 was used as the UV (365 & 385 nm), and visible (460 nm) light source for polymerization and actuation purposes. UV-vis absorption spectra and *cis–trans* isomerization kinetics were measured using Agilent Cary 60 spectrophotometer and the CoolLED pE-4000 as the excitation source. Photographs were taken, and movies recorded using a Canon 5D Mark III camera equipped with a 100 mm objective.

## Author contributions

The project was planned, and the material concept conceived jointly by H. G. and A. P.; R. N. conducted the experiments including LCE fabrication, characterization and analysis with the help of H. G.; R. N., H. G. and A. P. wrote the manuscript.

## Data availability

The authors declare that the data that supports the findings of this manuscript can be found in the ESI.† The raw data is available from the corresponding author upon request.

## Conflicts of interest

There are no conflicts to declare.

## Supplementary Material

TB-013-D4TB02228A-s001

TB-013-D4TB02228A-s002

TB-013-D4TB02228A-s003

## References

[cit1] Herbert K. M., Fowler H. E., McCracken J. M., Schlafmann K. R., Koch J. A., White T. J. (2022). Nat. Rev. Mater..

[cit2] Jiang Z. C., Liu Q., Xiao Y. Y., Zhao Y. (2024). Prog. Polym. Sci..

[cit3] Brannum M. T., Steele A. M., Venetos M. C., Korley L. S. T. J., Wnek G. E., White T. J. (2019). Adv. Opt. Mater..

[cit4] Hisano K., Kimura S., Ku K., Shigeyama T., Akamatsu N., Shishido A., Tsutsumi O. (2021). Adv. Funct. Mater..

[cit5] Zhang Y., Wang Z., Yang Y., Chen Q., Qian X., Wu Y., Liang H., Xu Y., Wei Y., Ji Y. (2020). Sci. Adv..

[cit6] Kuenstler A. S., Chen Y., Bui P., Kim H., DeSimone A., Jin L., Hayward R. C. (2020). Adv. Mater..

[cit7] Li K., Du C., He Q., Cai S. (2022). Ext. Mech. Lett..

[cit8] He Q., Wang Z., Wang Y., Minori A., Tolley M. T., Cai S. (2019). Sci. Adv..

[cit9] Zhang J., Guo Y., Hu W., Hao Soon R., Davidson Z. S., Sitti M. (2021). Adv. Mater..

[cit10] Pilz Da Cunha M., Debije M. G., Schenning A. P. H. J. (2020). Chem. Soc. Rev..

[cit11] Zhang Y., Wang Z., Yang Y., Chen Q., Qian X., Wu Y., Liang H., Xu Y., Wei Y., Ji Y. (2020). Sci. Adv..

[cit12] Nam S., Jung W., Shin J. H., Choi S. S. (2024). Light: Sci. Appl..

[cit13] Prévôt M. E., Andro H., Alexander S. L. M., Ustunel S., Zhu C., Nikolov Z., Rafferty S. T., Brannum M. T., Kinsel B., Korley L. T. J., Freeman E. J., McDonough J. A., Clements R. J., Hegmann E. (2018). Soft Matter.

[cit14] Jiang J., Dhakal N. P., Guo Y., Andre C., Thompson L., Skalli O., Peng C. (2020). Adv. Healthcare Mater..

[cit15] Wani O. M., Zeng H., Priimagi A. (2017). Nat. Commun..

[cit16] Liu X., Wei R., Hoang P. T., Wang X., Liu T., Keller P. (2015). Adv. Funct. Mater..

[cit17] Ji Y., Huang Y. Y., Rungsawang R., Terentjev E. M. (2010). Adv. Mater..

[cit18] Sartori P., Yadav R. S., del Barrio J., DeSimone A., Sánchez-Somolinos C. (2024). Adv. Sci..

[cit19] Ryabchun A., Li Q., Lancia F., Aprahamian I., Katsonis N. (2019). J. Am. Chem. Soc..

[cit20] Guillen Campos J., Tobin C., Sandlass S., Park M., Wu Y., Gordon M., Read de Alaniz J. (2024). Adv. Mater..

[cit21] Jin B., Yang S. (2023). Adv. Funct. Mater..

[cit22] Gelebart A. H., Mulder D. J., Vantomme G., Schenning A. P. H. J., Broer D. J. (2017). Angew. Chem., Int. Ed..

[cit23] Lahikainen M., Zeng H., Priimagi A. (2018). Nat. Commun..

[cit24] Lan R., Shen W., Yao W., Chen J., Chen X., Yang H. (2023). Mater. Horiz..

[cit25] Abdelrahman M. K., Wagner R. J., Kalairaj M. S., Zadan M., Kim M. H., Jang L. K., Wang S., Javed M., Dana A., Singh K. A., Hargett S. E., Gaharwar A. K., Majidi C., Vernerey F. J., Ware T. H. (2024). Nat. Mater..

[cit26] Hu Z., Fang W., Li Q., Feng X. Q., Lv J. A. (2020). Nat. Commun..

[cit27] Liu Z., Bisoyi H. K., Huang Y., Wang M., Yang H., Li Q. (2022). Angew. Chem., Int. Ed..

[cit28] Zeng H., Zhang H., Ikkala O., Priimagi A. (2020). Matter.

[cit29] Liang H., Zhang S., Liu Y., Yang Y., Zhang Y., Wu Y., Xu H., Wei Y., Ji Y. (2023). Adv. Mater..

[cit30] Saed M. O., Gablier A., Terentjev E. M. (2022). Chem. Rev..

[cit31] Lugger S. J. D., Houben S. J. A., Foelen Y., Debije M. G., Schenning A. P. H. J., Mulder D. J. (2022). Chem. Rev..

[cit32] Lugger S. J. D., Ceamanos L., Mulder D. J., Sánchez-Somolinos C., Schenning A. P. H. J. (2023). Adv. Mater. Tech..

[cit33] Guo H., Liang C., Ruoko T. P., Meteling H., Peng B., Zeng H., Priimagi A. (2023). Angew. Chem., Int. Ed..

[cit34] Lu X., Ambulo C. P., Wang S., Rivera-Tarazona L. K., Kim H., Searles K., Ware T. H. (2021). Angew. Chem., Int. Ed..

[cit35] Broer D. J., Bastiaansen C. M. W., Debije M. G., Schenning A. P. H. J. (2012). Angew. Chem., Int. Ed..

[cit36] Harris K. D., Bastiaansen C. W. M., Lub J., Broer D. J. (2005). Nano Lett..

[cit37] Wani O. M., Verpaalen R., Zeng H., Priimagi A., Schenning A. P. H. J. (2019). Adv. Mater..

[cit38] Guo H., Ruoko T. P., Zeng H., Priimagi A. (2024). Adv. Funct. Mater..

[cit39] De Haan L. T., Verjans J. M. N., Broer D. J., Bastiaansen C. W. M., Schenning A. P. H. J. (2014). J. Am. Chem. Soc..

[cit40] Van Heeswijk E. P. A., Kloos J. J. H., Grossiord N., Schenning A. P. H. J. (2019). J. Mater. Chem. A.

[cit41] Ryabchun A., Lancia F., Nguindjel A. D., Katsonis N. (2017). Soft Matter.

[cit42] Ware T. H., McConney M. E., Wie J. J., Tondiglia V. P., White T. J. (2015). Science.

[cit43] Zou W., Lin X., Terentjev E. M., Zou W., Lin X., Terentjev E. M. (2021). Adv. Mater..

[cit44] Yoon H. H., Kim D. Y., Jeong K. U., Ahn S. K. (2018). Macromolecules.

[cit45] Ikeda T., Mamiya J. I., Yu Y. (2007). Angew. Chem., Int. Ed..

[cit46] Yu Y., Nakano M., Ikeda T. (2003). Nature.

[cit47] Wang J., Huang S., Zhang Y., Liu J., Yu M., Yu H. (2021). ACS Appl. Mater. Interfaces.

[cit48] Liu G., Guan C., Xia H., Guo F., Ding X., Peng Y. (2006). Macro. Rapid Commun..

[cit49] Ware T., Hearon K., Lonnecker A., Wooley K. L., Maitland D. J., Voit W. (2012). Macromolecules.

[cit50] Song Y., Chen Y., Chen R., Zhang H., Shi D., Wang Y., Dong W., Ma P., Zhao Y. (2021). ACS Appl. Polym. Mater..

[cit51] Lewis K. L., Herbert K. M., Matavulj V. M., Hoang J. D., Ellison E. T., Bauman G. E., Herman J. A., White T. J. (2023). ACS Appl. Mater. Interfaces.

