# Supplementary material for: Hydrogen-bonded multi-mode liquid crystal elastomer actuators
Source: J Mater Chem B. 2024 Dec 18;13(5):1704–11. doi: 10.1039/d4tb02228a (PMC11696861; doi:10.1039/d4tb02228a)
Supplement: TB-013-D4TB02228A-s003 [file TB-013-D4TB02228A-s003.pdf]

Supplementary Information (SI) for Journal of Materials Chemistry B.
This journal is © The Royal Society of Chemistry 2025

Supporting Information for

Hydrogen-bonded Multi-Mode Liquid Crystal Elastomer Actuators

Roshan Nasare, Hongshuang Guo, and Arri Priimagi**

Roshan Nasare, Dr. H. Guo, Prof. A. Priimagi,

Smart Photonic Materials, Faculty of Engineering and Natural Sciences, Tampere University, Tampere, P.O. Box 541, FI-33101, Finland.

E-mail: hongshuang.guo@tuni.fi (0468415232), arri.priimagi@tuni.fi (0445150300)

Order parameter

Before and after base treatment

Order parameter have been checked for the LCE films before and after base treatment of 20 um thickness. The absorption spectra parallel and perpendicular to the molecular director are shown in **Fig. S1.** Sp was found to be 0.47 for non-base treated sample, and 0.45 for the base treated sample, with the help of **eq. 1**. Absorption is average data in wavelength range 405 – 425 nm with 20 um film.

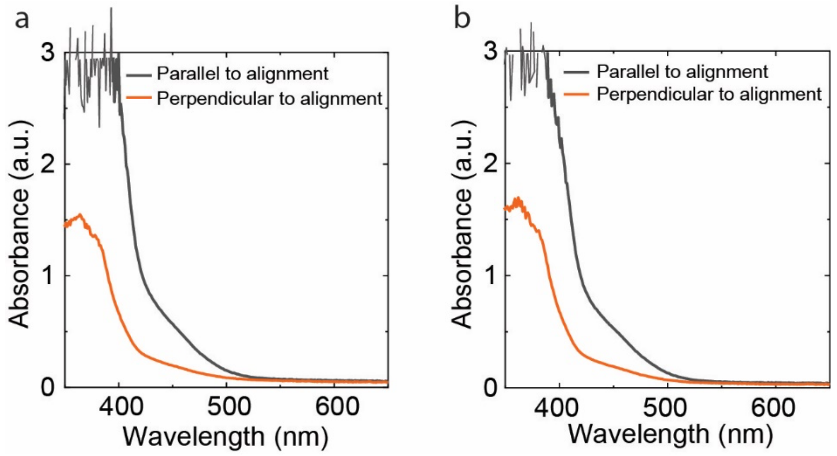

Fig S1. Polarized absorption spectra of a homogeneously aligned LCE for a) before, and b) after base treated film with thickness 20 um.

The order parameter, S, can be calculated as

$$S = \frac{(A\| - A\perp)}{(A\| + 2\,A\perp)} \qquad \text{...... (eq. 1)}$$

where A‖ and A⊥ are the measured absorbance values with light polarized parallel and perpendicular to the LC alignment, respectively.

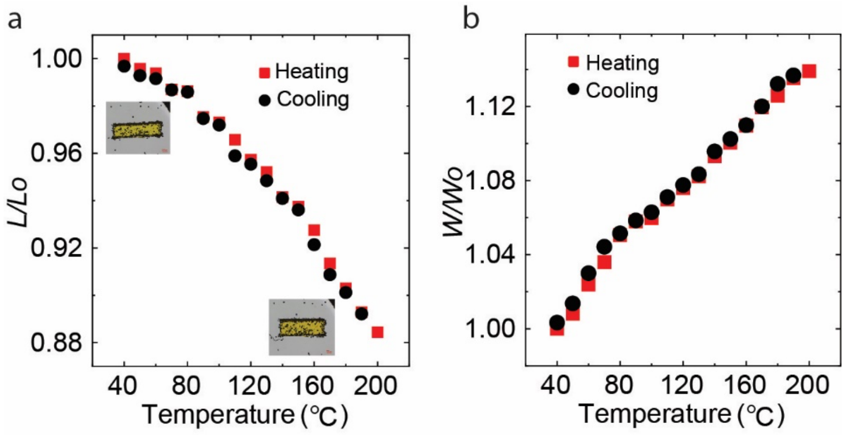

Fig S2. Heat-induced deformability in a planar-aligned LCE sample (a) along and (b) perpendicular to the alignment direction.

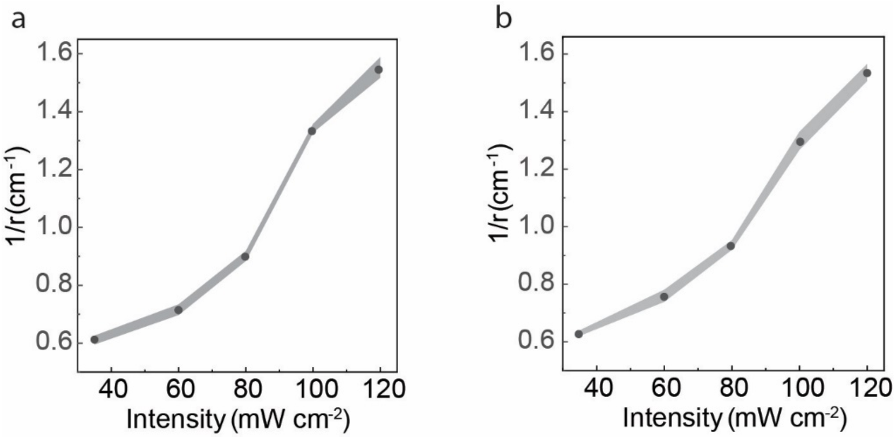

Fig S3. Change in bending curvature on increasing light intensity, a) light source right side, b) light source left side.

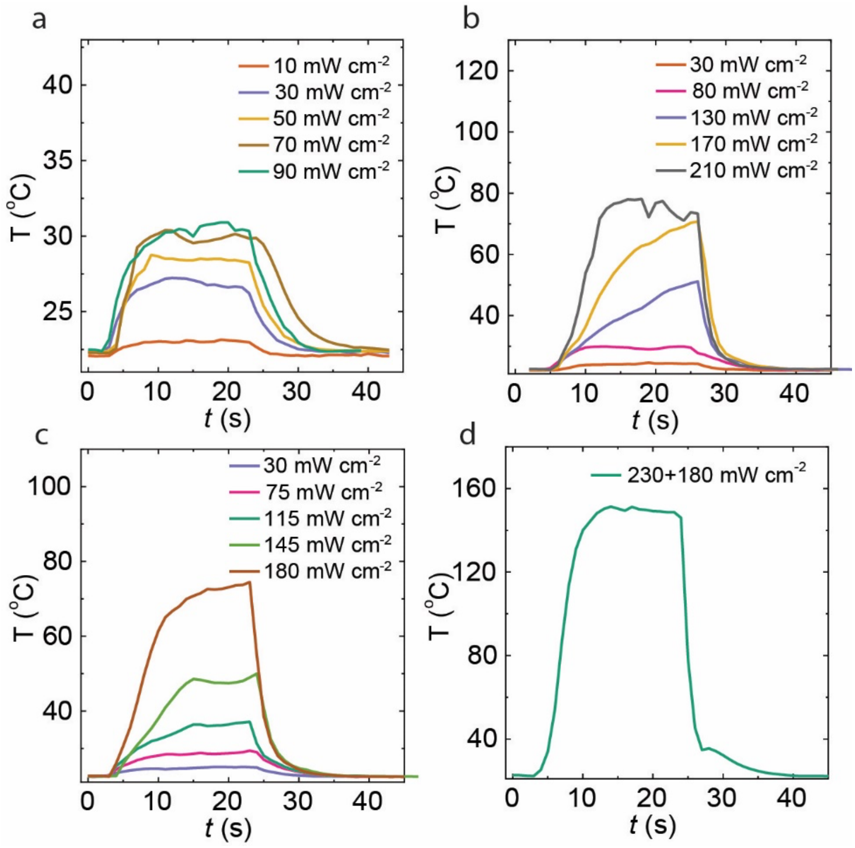

Figure S4: Temperature kinetic curve of non-base treated LCE strip upon different light intensity for wavelength: (a) 365nm, (b) 385nm, (c) 460nm, (d) 385nm+460nm.

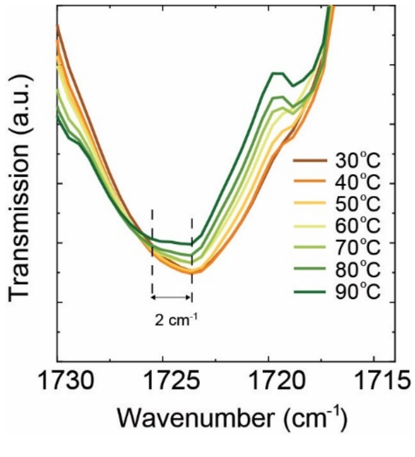

Figure S5: FTIR spectra of the LCE at different temperatures upon heating from 30 to 90 °C.

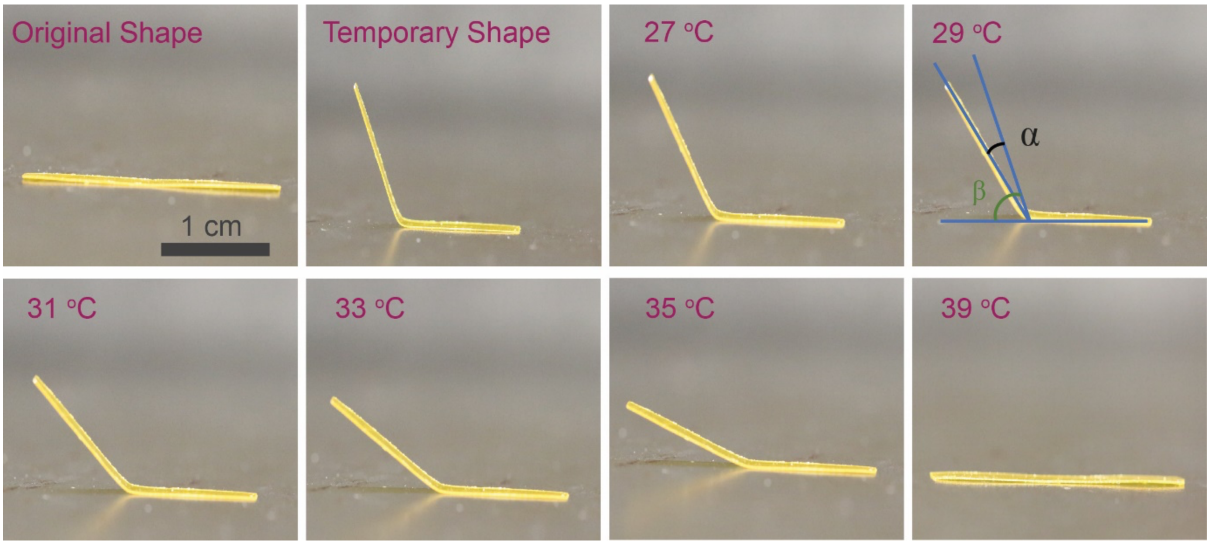

Figure S6: Photographs for Shape recovery ratio of non-activated LCE film at different temperatures. The recovery ratio was defined by α/β x 100%.

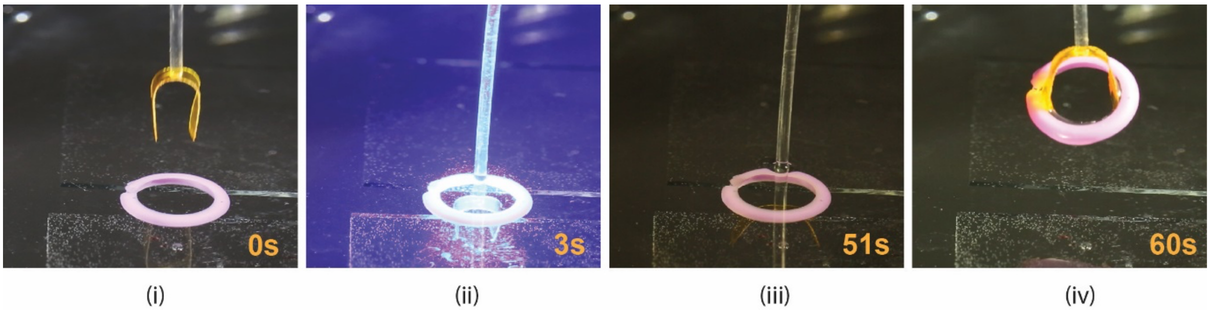

Figure S7: Light-controllable gripper under water based on the shape memory effect: (i) LCE is programmed to pass through a circular object. (ii) 385 + 460 nm (150 + 100 mW cm^{-2}, 50 secs) light irradiation induces a photothermal effect on the LCE film. (iii) Due to the photothermal effect, the LCE returns to its original shape. (iv) The object is lifted off.

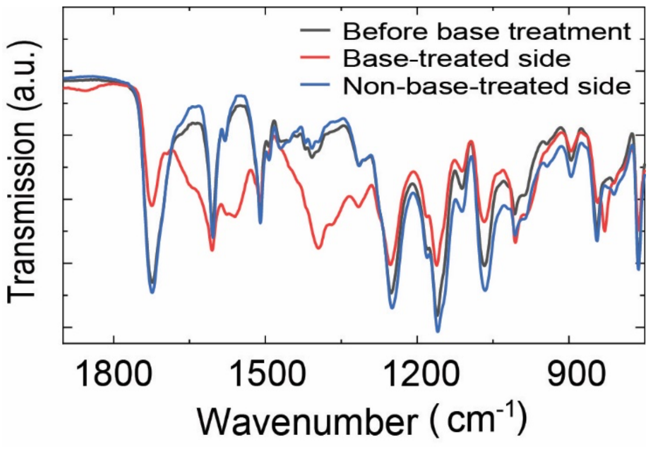

Figure S8: FTIR spectra of the film before and after activation.

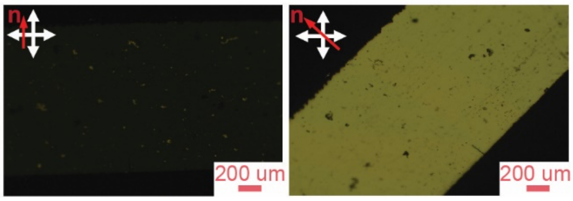

Figure S9: Polarized optical microscopy (POM) images of the base treated LCE at 0° and 45° between the molecular director and the polarizer/analyzer, demonstrating uniaxial molecular alignment.

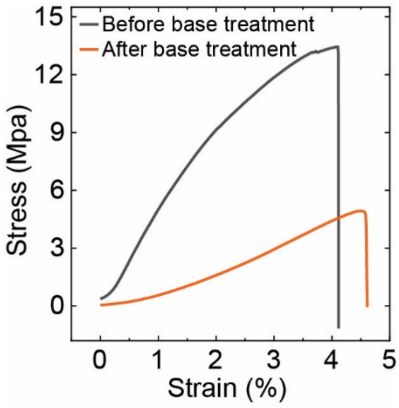

Figure S10: Tensile stress–strain curves of before and after base treated film.

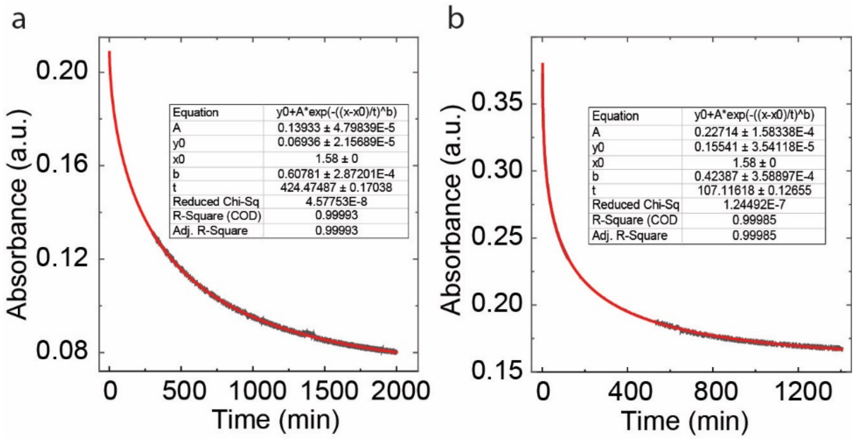

Figure S11: Exponential fit graph for the half-life of LCE (a) before and (b) after base treatment.

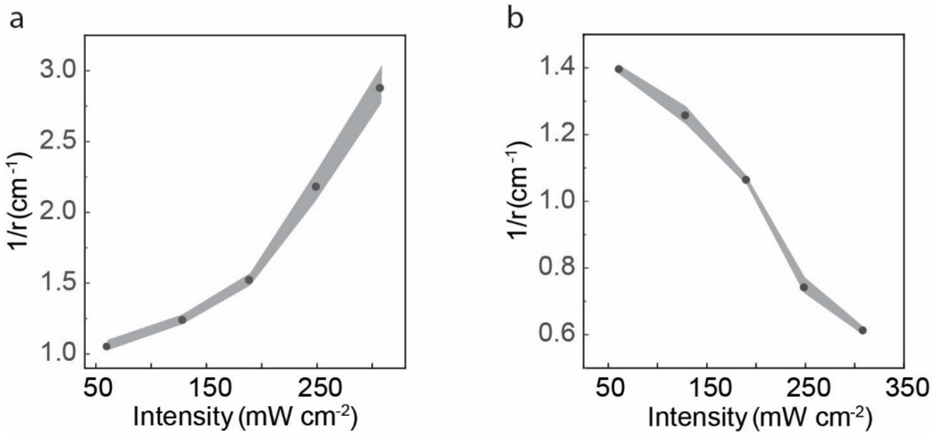

Figure S12. Change in bending curvature on increasing light intensity, (a) light source towards base treated side, (b) light source towards non-base treated side.

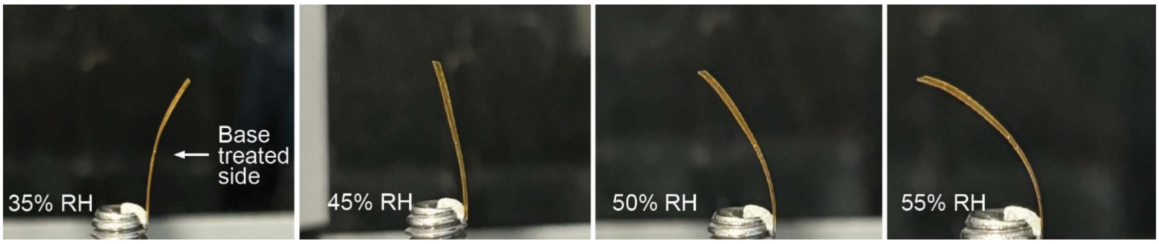

Figure S13. Deformation of base treated LCE in response to humidity stored 4 months in ambient conditions**.**

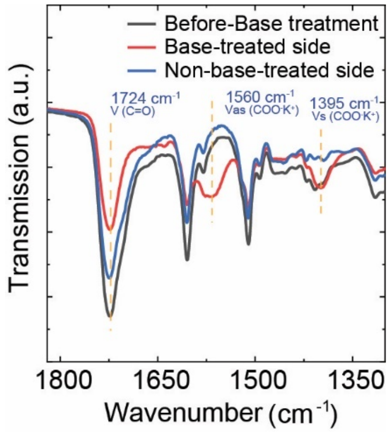

Figure S14. FTIR spectra of base-treated LCE after 4 months of storage in ambient conditions.

Movie S1. Light-controllable gripper in air based on the shape memory effect.

Movie S2. Light-controllable gripper under water based on the shape memory effect.
